# Cross-Cultural Adaptation and Psychometric Evaluation of Greek Nutrition for Sport Knowledge Questionnaire (NSKQ-Gr): Evidence from Classical Test Theory and Rasch Modeling

**DOI:** 10.3390/sports14080331

**Published:** 2026-08-03

**Authors:** Eirini Koidou, Fani Thoma, Christina F. Dolopikou, Sousana K. Papadopoulou, Constantinos Giaginis, Vassilis Barkoukis, Julie Vaiopoulou

**Affiliations:** 1 School of Physical Education and Sports Science, Faculty of Physical Education and Sport Sciences, Aristotle University of Thessaloniki, 62122 Serres, Greece; fannithoma@gmail.com (F.T.); cdolopik@phed-sr.auth.gr (C.F.D.); 2 Department of Nutritional Sciences & Dietetics, Faculty of Health Sciences, International Hellenic University, 57001 Thessaloniki, Greece; 3 Department of Food Science and Nutrition, School of Environment, University of the Aegean, 81400 Lemnos, Greece; cgiaginis@aegean.gr; 4 School of Physical Education and Sports Science, Faculty of Physical Education and Sport Sciences, Aristotle University of Thessaloniki, 54124 Thessaloniki, Greece; bark@phed.auth.gr; 5 Department of Education, University of Nicosia, Nicosia 2417, Cyprus; vaiopouloucp@unic.ac.cy

**Keywords:** sports nutrition knowledge, coaches, questionnaire validation, psychometrics

## Abstract

**Background/Objectives**: Valid and culturally adapted instruments are essential for accurately assessing sports nutrition knowledge among coaches, given their influential role in shaping athletes’ dietary behaviors and performance outcomes. This study aimed to translate, culturally adapt, and evaluate the psychometric properties of the Nutrition for Sport Knowledge Questionnaire (NSKQ) for Greek certified coaches, while also examining knowledge levels and associated demographic factors. **Methods**: A cross-sectional study was conducted among 514 certified coaches representing various sports disciplines. The translation followed a standardized forward–backward procedure with expert panel review to ensure cultural relevance. The psychometric properties of the NSKQ-Gr were evaluated using complementary Classical Test Theory (CTT) and Rasch approaches. Item difficulty, item discrimination, Kuder–Richardson Formula 20 (KR-20), McDonald’s omega, and confirmatory factor analyses (CFAs) using the WLSMV estimator for dichotomous items were performed. Test–retest reliability was examined via Pearson’s correlation coefficient and intraclass correlation coefficient (ICC). Statistical analyses explored associations between nutrition knowledge and demographic and professional characteristics. **Results**: Item discrimination varied across domains; the strongest performance was observed for the Alcohol domain and the weakest for Weight Management and Sports Nutrition. KR-20 and McDonald’s omega indicated moderate internal consistency for most domains, apart from Weight Management and Sports Nutrition, the reliability of which was lower. The temporal stability was excellent (ICC = 0.998). CFA findings were heterogeneous across domains, whereas Rasch analyses showed generally acceptable item-level fit. Most coaches demonstrated average (56.8%) or poor (32.3%) sports nutrition knowledge. Good or excellent knowledge was demonstrated by only 10.9% of the participants. Knowledge scores differed according to age, certification pathway, previous athletic experience, and several nutrition-related coaching practices. **Conclusions**: The NSKQ-Gr provides a useful multidomain instrument for assessing sports nutrition knowledge among Greek coaches. The present study extends previous validation work by providing complementary evidence from CTT, CFA, and Rasch modeling, thereby offering a more comprehensive psychometric evaluation of the NSKQ-Gr. The findings indicate that the NSKQ-Gr is a multidomain knowledge assessment instrument suitable for Greek coaches. Future validation studies are encouraged to further refine its psychometric properties.

## 1. Introduction

Athletic performance is a multifactorial phenomenon resulting from the interaction of multiple biological systems within specific training and environmental contexts. Adequate nutrition and healthy eating habits directly impact energy levels, recovery, endurance, injury prevention, and mental clarity, all of which are fundamental for peak athletic performance. In addition, appropriate nutritional strategies help prevent overtraining and burnout while supporting optimal performance during both training and competition. Although previous studies have shown that athletes, independently of age, sex, level, or type of sport, have sub-optimal energy and carbohydrate intake, high intake of fats, insufficient mineral and vitamin intake, and underhydration. Similarly, the prevalence of disordered eating, relative energy deficiency in sport (REDS), and overtraining syndrome is rising. 

Several studies have demonstrated that coaches’ behaviors, attitudes, and nutrition knowledge substantially influence athletes’ health and sport performance. Contemporary coaching adopts a holistic, athlete-centered approach in which coaches assume leadership roles by fostering discipline, accountability, perseverance, healthy lifestyle behaviors, and appropriate nutrition practices [1,2,3,4]. Zinn et al. [5] reported that 43% of the athletes turned to their coach when they had questions about such topics, although nutrition knowledge was not related to better dietary intakes. Therefore, coaches represent an important source of nutritional information, and they can help athletes recognize the importance of diet for performance and recovery while collaborating with nutritionists. Previous studies have shown that coaches’ knowledge in this area directly impacts the quality of advice they provide to athletes, which can influence dietary choices, performance outcomes, and long-term health [6]. However, research consistently indicates that many coaches lack basic and adequate nutritional knowledge. Most studies report that coaches demonstrate moderate levels of nutrition knowledge [5,7,8,9,10,11,12], whereas several others have described their knowledge as poor and concluded that many coaches lack the nutritional background required to provide evidence-based dietary advice to athletes [13,14,15,16,17]. Only two studies involving coaches of elite athletes reported adequate levels of nutrition knowledge. Specifically, Aka [18] compared coaches from professional and amateur soccer teams and found that coaches working in the Super League demonstrated greater nutrition knowledge than those coaching in lower divisions. Similarly, Tesema and Mohan [11] reported that coaches whose teams finished in the top three league positions achieved higher nutrition knowledge scores than coaches whose teams ranked in the bottom three.

Several studies have also examined the association between coaches’ characteristics and nutrition knowledge. No significant association has been observed between coaches’ sex and nutrition management practices, willingness to provide nutrition advice, or awareness of nutrition-related issues associated with diet and weight loss [17,18,19]. However, Heikkilä et al. [12] reported that female coaches achieved significantly higher nutrition knowledge scores than male coaches. 

Regarding coaching experience, two studies reported that more experienced coaches possess better nutritional knowledge [20,21], whereas other studies have found no significant differences according to the years of coaching experience [16,17,19,22]. In contrast, higher educational attainment has consistently been associated with greater nutrition knowledge [7,19,23,24].

Studies have suggested that coaches who provide nutrition advice to athletes generally exhibit higher levels of nutrition knowledge [5,9]. One study reported that coaches training female athletes had slightly higher levels of knowledge [25], whereas another study reported that coaches of female athletes were more knowledgeable about weight loss/gain topics, whereas those coaching male athletes were better informed on hydration and muscle mass gain [21]. Despite these findings, many coaches lack formal nutrition education [9,14,22], and only a small proportion of coaches seek advice from professionals. Studies investigating whether nutrition knowledge differs according to sport type have generally reported no significant differences. Couture et al. [19] found no differences between coaches of aesthetic and non-aesthetic sports, and similar findings were reported in studies involving coaches from tennis, gymnastics, judo, swimming, basketball, and football [9,22].

Most studies support the idea that coaches with sufficient nutritional knowledge and appropriate nutrition education can positively influence athletes’ dietary behaviors, performance, health, and career longevity. 

To evaluate the nutritional knowledge of coaches, most studies used questionnaires specifically designed for the needs of each respective study, with the assistance of dietitians. These questionnaires covered various aspects of nutrition, such as macronutrients and micronutrients, hydration, as well as the coaches’ opinions and practices (recommendations for weight loss, weighing, etc.), which shape their attitudes towards nutrition. More recently, the Nutrition for Sport Knowledge Questionnaire (NSKQ) [26] has been used to evaluate general and sports nutritional knowledge in athletes and coaches. 

The original NSKQ was developed via a combination of Classical Test Theory and Rasch measurement approaches, while evidence for the questionnaire’s internal structure and measurement properties was also provided. Previous translated versions [27,28] have reported encouraging evidence regarding reliability and validity. Nevertheless, more comprehensive evaluations that integrate item-level analyses, factorial validity, and Rasch modeling remain limited. Thus, the need arises for further research providing additional psychometric evidence.

In this context, the aim of the present study was to translate, culturally adapt, and comprehensively evaluate the psychometric properties of the Greek version of the Nutrition for Sport Knowledge Questionnaire (NSKQ-Gr) for certified Greek coaches. Reliability estimation, Classical Test Theory, CFA, and Rasch approaches were implemented, following the notion that the complementary finding would provide stronger evidence for the psychometric properties of the NSKQ-Gr than would be obtained from any single analytical approach. Finally, sports nutrition knowledge levels and their associations with demographic and professional characteristics were investigated.

## 2. Materials and Methods

### 2.1. Study Design 

This was a cross-sectional study conducted to translate, culturally adapt, and evaluate the psychometric properties of the NSKQ-Gr. In addition, the study also assessed sports nutrition knowledge among Greek certified coaches and examined its associations with selected sociodemographic and professional characteristics. 

### 2.2. Ethical Approval 

Ethical approval was obtained from the Bioethics Committee of Aristotle University of Thessaloniki, specifically the Ethics Committee of the School of Physical Education and Sport Sciences (Serres), under protocol number ERC-014/2022.

### 2.3. Procedure 

Participants were informed about the purpose of the study, the study procedures, and the estimated time required to complete the questionnaire before providing written informed consent. Participants who agreed to complete the questionnaire a second time were informed about the follow-up procedure. Data were collected between October 2023 and January 2024. Although the questionnaire was initially completed by a larger number of coaches, only those who met the predefined inclusion criteria were included in the final analysis (*n* = 514). Of these, 103 coaches completed the questionnaire a second time after a 10-day interval.

### 2.4. Participants 

A total of 514 certified coaches (308 males and 206 females; mean age: 34.88 ± 9.09 and 35.41 ± 9.23 years, respectively) participated in the study. All participants were certified coaches working with adolescent athletes aged 14–17 years in organized sports clubs across Greece. The sample therefore represents coaches primarily involved in youth club sports. Participants were recruited from sports clubs throughout the country, providing broad geographical representation. The participating coaches represented a broad range of individual and team sports. Specifically, 108 coaches (21.0%) coached martial arts, 74 (14.4%) basketball, 72 (14.0%) soccer, 66 (12.8%) volleyball, 52 (10.1%) track and field, 46 (8.9%) swimming, 26 (5.1%) boxing, 22 (4.3%) tennis, 20 (3.9%) wheelchair basketball, 18 (3.5%) gymnastics, 6 (1.2%) badminton, and 4 (0.8%) handball. These characteristics indicate that the study sample reflects certified coaches working in organized youth sports clubs across Greece, representing a broad range of sporting disciplines.

### 2.5. Measures

The questionnaire used in the present study was the full-length version of the Nutrition for Sport Knowledge Questionnaire (NSKQ), consisting of 87 scored questions. The NSKQ full-length was developed in 2017 by Trakman et al. [26]. According to the authors, the NSKQ was based on current sports nutrition recommendations and is comprehensive. It was developed in collaboration with a panel of international sports dietitians and validated through a rigorous methodology, incorporating both Classical Test Theory and Rasch analysis. The questionnaire contains 87 scored items across six nutrition domains. [weight management (12), macronutrients (30), micronutrients (13), sports nutrition (12), supplementation (12), alcohol (8)]. The questionnaire includes multiple-choice items with a single correct response. Each correct answer is awarded one point, whereas incorrect and “Not sure” responses receive zero points; no negative marking is applied. The total score is converted to a percentage, and nutrition knowledge is classified according to the original scoring system as poor (0–49%), average (50–65%), good (66–75%), or excellent (76–100%).

With the kind permission of Gina Trakman, an author of the questionnaire, the questionnaire was translated into Greek and then back into English by two bilingual translators following the methodology and practical guidelines by WHO and Degroot et al. [29,30]. A bilingual dietitian translated the questionnaire from Greek to English. A group of 10 scientists (5 were professors in Greek universities) with nutritional backgrounds in dieting and clinical and sport nutrition reviewed each question of the Greek version. All questions were kept, but specific foods were replaced with foods (with the same characteristics, e.g., nutritional value, food group, gr of nutrients) known and commonly consumed in Greece. Also, it utilized the metric system, which is commonly used in Greece. The outcome of the pretesting group resulted in minor improvements to the wording of certain questions to better align with Greek linguistic culture in relation to food and diet. 

### 2.6. Demographic Characteristics of the Participants

Data were collected through a self-report questionnaire using an online sample (the questionnaire is designed to be administered online). Participants provided self-reported information on sex, age, marital status, educational level, perceived financial status, and whether coaching constituted their primary source of income. Body mass index (BMI) was calculated from self-reported height and weight, and participants were subsequently classified as having normal weight, overweight, or obesity according to standard BMI categories. Participants also reported whether they had previously been competitive athletes, whether they provided nutrition advice to their athletes, the frequency with which they provided such advice, whether they routinely weighed their athletes, whether they considered themselves informed about sports nutrition, and their primary sources of sports nutrition information. Responses to these items were recorded using either dichotomous (yes/no) or multiple-choice response formats, as appropriate.

The average time required to complete the questionnaire was approximately 27 min during the first administration and 28 min during the second administration.

### 2.7. Statistical Analyses 

The analyses were conducted using IBM SPSS Statistics for Windows, version 29.0 (IBM Corp., Armonk, NY, USA), and R, version 4.3.2 (R Foundation for Statistical Computing, Vienna, Austria) [31]. Additional psychometric analyses were performed using the *lavaan* (version 0.6-21) [32], *psych* (version 2.6.3) [33], *eRm* (version 1.0-10) [34], and *TAM* (version 4.3-25) [35] packages.

Sociodemographic data were reported as absolute and relative frequencies; descriptive statistics were used for continuous variables and frequency analyses for categorical variables. Prior to the psychometric evaluation, data were examined for missing values and item variability. Normality was assessed by the Kolmogorov–Smirnov test. 

Item difficulty was calculated as the proportion of participants answering each dichotomously scored item correctly; lower values indicated more difficult items. Corrected item–total correlations were used to evaluate item discrimination. The correlations were calculated within each knowledge domain, with lower values indicating weaker item discrimination [35].

Kuder–Richardson Formula 20 (KR-20) and McDonald’s omega (ω), two reliability coefficients that are appropriate for dichotomously scored items [36,37], were used to assess internal consistency. Values ≥ 0.70 were considered indicative of acceptable internal consistency. 

Factorial validity was examined separately for each domain using confirmatory factor analysis (CFA). CFAs were estimated using the weighted least squares mean and variance adjusted (WLSMV) estimator appropriate for dichotomous indicators [38,39]. Model fit was evaluated using the *χ*^2^ statistic, Comparative Fit Index (CFI), Tucker–Lewis Index (TLI), Root Mean Square Error of Approximation (RMSEA), and Standardized Root Mean Square Residual (SRMR). Model fit was considered acceptable if CFI and TLI values ≥ 0.90 and RMSEA and SRMR values ≤ 0.08 [40].

Rasch analyses were performed separately for each domain using the dichotomous Rasch model [41]. Item difficulty parameters, infit and outfit mean-square statistics, Andersen likelihood-ratio tests, and Wright person–item maps were estimated. Infit and outfit values between 0.70 and 1.30 were considered indicative of acceptable item fit [42].

Test–retest reliability was assessed using the intraclass correlation coefficient (ICC) [43].

Pearson’s correlation coefficients were calculated to examine the relationships among the NSKQ domain scores and the total NSKQ score. Chi-square tests of independence were performed to examine the associations between nutrition knowledge categories and participants’ sex, as well as between nutrition knowledge categories and the type of sport coached.

Differences in sports nutrition knowledge (NK) scores according to participants’ demographic and professional characteristics were examined using one-way analysis of variance (ANOVA) with Bonferroni post hoc tests, the independent-samples *t*-test, or the Mann–Whitney U test, as appropriate. Analyses examining the associations between sports nutrition knowledge and demographic or professional characteristics were considered exploratory and conducted to identify potential factors associated with sports nutrition knowledge rather than test predefined primary hypotheses. All statistical significance was determined at a 95% confidence interval (CI), with a *p*-value threshold of ≤0.05.

## 3. Results

### 3.1. Coaches’ Anthropometric and Demographic Characteristics and Nutrition Knowledge

The anthropometric data of the coaches are presented in Table 1.

According to the NSKQ scores developed by Trakman et al. [26], 32.3% of coaches demonstrated poor nutrition knowledge, 56.8% had average knowledge, 8.6% had good knowledge, and 2.3% had excellent knowledge. Table 2 shows the results by sex and type of sport in relation to the level of nutritional knowledge.

### 3.2. Descriptive Statistics and Inter-Domain Correlations

Table 3 presents the descriptive statistics for the NSKQ-Gr domains and their correlations. Significant positive correlations (*r* = 0.338–0.536, *p* < 0.001) were observed among Weight Management, Macronutrients, Micronutrients, Sports Nutrition, and Nutritional Supplements. The Alcohol domain demonstrated significantly weaker associations with the remaining domains, showed only one correlation with Macronutrients (*r* = 0.153, *p* = 0.001); its correlations with every other domain, i.e., Weight Management (*r* = 0.081, *p* = 0.068), Micronutrients (*r* = 0.041, *p* = 0.357), Sports Nutrition (*r* = 0.051, *p* = 0.246), and Nutritional Supplements (*r* = 0.075, *p* = 0.091), were not statistically significant.

### 3.3. Psychometric Evaluation of the NSKQ-Gr

#### 3.3.1. Preliminary Data Screening 

Prior to the psychometric analyses, the dataset was screened for missing values, item variability, and redundant items. No missing responses were identified. NSKQ80 and NSKQ85 demonstrated zero variance, i.e., all 514 participants provided the same response. NSKQ82 and NSKQ86 had perfect redundancy (*r* = 1.00), indicating identical response patterns across all participants. These findings were considered during the subsequent psychometric evaluation.

#### 3.3.2. Item Analysis 

Item difficulty was calculated as the proportion of participants answering each item correctly; thus, lower percentages indicate more difficult items, whereas higher percentages indicate easier items. The proportion of correct responses ranged from 4.3% to 100.0% (M = 52.6%, SD = 23.9%), thus indicating substantial variability in item difficulty. Twelve items were answered correctly by more than 80% of participants and ten items were answered correctly by fewer than 20% of participants, demonstrating that the questionnaire contained both very easy and very difficult items. Table 4 summarizes the items with the lowest and highest proportions of correct responses. Complete item-level psychometric results are presented in Table A1.

Corrected item–total correlations were calculated separately within each NSKQ domain. At the domain level, mean item difficulty ranged from 39.4% for Nutritional Supplements to 79.7% for Alcohol, with the remaining domains demonstrating intermediate levels of difficulty. 

Item discrimination varied considerably across the questionnaire (Table 5). The Alcohol domain showed the strongest average discrimination (*M* = 0.357), with only one of the six analyzed items displaying a corrected item–total correlation below 0.20. Mean corrected item–total correlations were lower for Micronutrients *(M* = 0.236), Nutritional Supplements (*M* = 0.236), and Macronutrients (*M* = 0.208), with 7, 4, and 13 items, respectively, falling below 0.20. The weakest discrimination was observed for Weight Management (*M* = 0.109) and Sports Nutrition (*M* = 0.094), in which 10 of the 12 items in each domain had corrected item–total correlations below 0.20. These findings indicate substantial heterogeneity in item discrimination, particularly within the Weight Management and Sports Nutrition domains. Table 5 summarizes the mean item difficulty and the mean corrected item–total correlations for the six domains. 

#### 3.3.3. Internal Consistency 

Kuder–Richardson Formula 20 (KR-20) and McDonald’s omega (*ω*) were calculated to evaluate the internal consistency. Macronutrients demonstrated the highest internal consistency (KR-20 = 0.674, *ω* = 0.696), followed by Alcohol (KR-20 = 0.605, *ω* = 0.741). Moderate reliability estimates were observed for Micronutrients (KR-20 = 0.575, *ω* = 0.616) and Nutritional Supplements (KR-20 = 0.570, *ω* = 0.594). Lower internal consistency was found for the Weight Management (KR-20 = 0.333, *ω* = 0.326) and Sports Nutrition (KR-20 = 0.306, *ω* = 0.284) domains.

#### 3.3.4. Confirmatory Factor Analysis

To examine the proposed factorial structure of the NSKQ-Gr, one-factor confirmatory factor analyses (CFAs) were estimated separately for each knowledge domain using the WLSMV estimator for dichotomous items. The results suggested that model fit varied across the six knowledge domains. Most domains did not have a good fit to the theoretical model: Weight Management (*χ^2^*(54) = 239.25, CFI = 0.496, TLI = 0.384, RMSEA = 0.082, 95% CI [0.071, 0.092], SRMR = 0.148), Macronutrients (*χ^2^*(405) = 1559.47, CFI = 0.375, TLI = 0.328, RMSEA = 0.075, 95% CI [.071, 0.078], SRMR = 0.165), Micronutrients (*χ^2^*(65) = 602.58, CFI = 0.362, TLI = 0.234, RMSEA = 0.127, 95% CI [.118, 0.136], SRMR = 0.218), Sports Nutrition (*χ^2^*(54) = 201.06, CFI = 0.455, TLI = 0.333, RMSEA = 0.073, 95% CI [0.062, 0.084], SRMR = 0.166), Nutritional Supplements (*χ^2^*(54) = 327.85, CFI = 0.628, TLI = 0.545, RMSEA = 0.099, 95% CI [.089, 0.110], SRMR = 0.149). The Alcohol domain demonstrated acceptable fit (*χ^2^*(5) = 39.24, CFI = 0.983, TLI = 0.966, RMSEA = 0.116, 95% CI [.084, 0.150], SRMR = 0.252). Standardized factor loadings were heterogeneous across domains, and several items had weak or negative loadings. The CFA findings are interpreted alongside the Classical Test Theory and Rasch results to provide a complementary evaluation of the internal structure of the NSKQ-Gr.

#### 3.3.5. Rasch Analysis

Rasch analyses were conducted separately for each NSKQ-Gr domain. Item difficulty parameters covered a broad range across the questionnaire, indicating that the domains included both relatively easy and difficult items. Item fit was evaluated using infit and outfit mean-square statistics; acceptable values ranged between 0.70 and 1.30. Detailed item-level Rasch fit statistics are presented in Table A1.

For Weight Management, mean infit and outfit values were 0.965 and 0.978, respectively. All items demonstrated acceptable infit, although NSKQ10 showed a moderately elevated outfit value of 1.397. The Andersen likelihood-ratio test was significant (*χ^2^*(11) = 117.58, *p* < 0.001).

For Macronutrients, mean infit was 0.992 and mean outfit was 0.994. Most items exhibited acceptable fit. NSKQ15 showed an outfit value of 1.339, while NSKQ28 demonstrated the largest deviation, with an outfit value of 1.502. The Andersen test was significant (*χ^2^*(29) = 360.41, *p* < 0.001).

For Micronutrients, mean infit and outfit values were 0.921 and 0.928, respectively. Item fit was generally satisfactory, although NSKQ53 showed a low outfit value of 0.641, suggesting overfit. The Andersen test was significant (*χ^2^*(12) = 69.22, *p* < 0.001).

For Sports Nutrition, mean infit was 0.929 and mean outfit was 0.942. NSKQ56 exhibited elevated outfit of 1.451, whereas NSKQ58 showed low outfit of 0.643. The remaining items were within the predefined fit range. The Andersen test was significant (*χ^2^*(11) = 91.03, *p* < 0.001).

For Nutritional Supplements, mean infit and outfit values were 0.978 and 0.942, respectively. All items demonstrated infit and outfit values within the acceptable range. Nevertheless, the Andersen test was significant (*χ^2^*(11) = 102.73, *p* < 0.001).

The Alcohol domain showed less satisfactory item functioning. Mean infit was 0.825 and mean outfit was 0.936, but the observed ranges were comparatively wide, from 0.608 to 1.408 for infit and from 0.408 to 1.894 for outfit. NSKQ81 showed elevated outfit, NSKQ82 and NSKQ86 demonstrated identical low fit values, NSKQ83 and NSKQ87 showed overfit, and NSKQ84 exhibited underfit. The Andersen likelihood-ratio test could not be computed for this domain because an insufficient number of appropriate response patterns remained after sample splitting.

Summing up, most items in the first five domains displayed acceptable individual Rasch fit, with only a small number showing moderate deviations. However, significant Andersen likelihood-ratio tests indicated that the Rasch model assumptions were not fully satisfied at the domain level. The Alcohol domain presented additional limitations because of two zero-variance items and the identical response patterns of NSKQ82 and NSKQ86.

#### 3.3.6. Test–Retest Reliability and Categorical Agreement

The NSKQ-Gr demonstrated excellent test–retest reliability. The overall intraclass correlation coefficient (ICC) was 0.998 (95% CI [0.997, 0.999]), indicating almost perfect temporal stability. At the domain level, ICC values ranged from 0.942 to 1.000, demonstrating excellent reproducibility across all six knowledge domains. Specifically, ICCs were 0.967 (95% CI [0.797, 0.971]) for Weight Management, 1.000 for Macronutrients, 1.000 for Micronutrients, 1.000 for Sports Nutrition, 0.942 (95% CI [0.776, 0.976]) for Nutritional Supplements, and 1.000 for Alcohol.

Paired samples *t*-tests revealed no significant differences between the two measurement points (*t*(102) = 0.168, *p* = 0.433). Also, test–retest reliability demonstrated Pearson correlation *r* = 0.993, and the inter-rater agreement demonstrated a moderate agreement between the two measurement points (*k* = 0.755, *p* < 0.001).

### 3.4. Associations Between Nutritional Knowledge and Participant Characteristics 

Associations between nutritional knowledge and participant characteristics were examined by comparing the distribution of sports nutrition knowledge categories across sex and sport type (Table 6). A statistically significant association was observed between sex and nutrition knowledge category (*χ^2^*(3, *N* = 514) = 28.49, *p* < 0.001), with a small-to-moderate effect size (Cramer’s *V* = 0.235). Specifically, male coaches were more frequently classified as having good nutrition knowledge than female coaches (11.0% vs. 4.9%), whereas female coaches were more frequently classified as having poor (37.9% vs. 28.6%) and excellent (5.8% vs. 0.0%) nutrition knowledge.

In contrast, nutrition knowledge categories did not differ significantly according to sport type (individual vs. team sports) (*χ^2^*(3, *N* = 514) = 2.94, *p* = 0.401, Cramer’s *V* = 0.076), indicating no meaningful association between the type of sport coached and sports nutrition knowledge.

The associations between coaches’ demographic and professional characteristics and total NSKQ scores are presented in Table 7.

A statistically significant effect of age on sports nutrition knowledge was observed (*F*(3, 510) = 3.839, *p* = 0.010, η^2^_p_ = 0.022). Bonferroni post hoc comparisons indicated that coaches aged 53–62 years (M = 49.87, SD = 4.17) scored significantly higher than those aged 22–32 years (M = 44.67, SD = 9.37), whereas no significant differences were found among the remaining age groups.

Coaches who obtained their professional certification through the Department of Physical Education and Sport Science (DPESS) achieved significantly higher NSKQ scores than coaches certified through other pathways (M = 47.08, SD = 8.37 vs. M = 43.44, SD = 11.05, Mann–Whitney *U* = 25,106, *p* < 0.001, *r* = 0.15). Similarly, coaches with previous athletic experience demonstrated significantly higher nutrition knowledge than those without such experience (M = 46.18, SD = 9.83 vs. M = 40.95, SD = 3.98, Mann–Whitney *U* = 5586, *p* < 0.001, *r* = 0.20).

No statistically significant differences in total NSKQ scores were observed according to sex (*t*(512) = 1.095, *p* = 0.274, Cohen’s *d* = 0.10, BMI category, *F*(3, 510) = 0.192, *p* = 0.902, η^2^_p_ = 0.001), coaching experience (*F*(2, 511) = 0.950, *p* = 0.388, η^2^_p_ = 0.007), level of previous athletic competition (*F*(2, 511) = 1.815, *p* = 0.164, η^2^_p_ = 0.008), or whether coaching constituted the participants’ primary occupation (Mann–Whitney *U* = 32,022, *p* = 0.596, *r* = 0.02).

### 3.5. Nutritional Knowledge and the Provision of Nutritional Counseling to Athletes

The coaches also answered questions regarding how they managed nutrition-related issues with their athletes. 

The associations between coaches’ nutrition-related practices and total NSKQ-Gr scores are presented in Table 8. Coaches who reported providing nutritional information to their athletes achieved significantly higher NSKQ-Gr scores than those who did not (M = 46.75, SD = 8.60 vs. M = 37.26, SD = 12.93, Mann–Whitney *U* = 6834, *p* < 0.001, *r* = 0.24).

The frequency with which coaches provided nutritional advice was also significantly associated with nutrition knowledge (*F*(2, 511) = 15.63, *p* < 0.001, η^2^_p_ = 0.065). Bonferroni post hoc comparisons indicated that coaches providing nutritional advice 1–2 times per week (M = 50.84, SD = 9.80) achieved significantly higher NSKQ-Gr scores than those providing advice 1–2 times per month (M = 45.82, SD = 7.70; *p* < 0.001, 95% CI [2.75, 7.30]) and those providing advice only if asked (M = 46.57, SD = 8.50; *p* < 0.001, 95% CI [2.45, 9.32]). No significant difference was observed between the latter two groups (*p* > 0.05).

Similarly, coaches who reported weighing their athletes demonstrated significantly higher nutrition knowledge than those who did not (M = 47.86, SD = 9.14 vs. M = 43.41, SD = 9.54, Mann–Whitney *U* = 24,030, *p* < 0.001, *r* = 0.23).

Finally, coaches who reported being informed about nutrition-related issues scored significantly higher than those who did not (M = 46.12, SD = 9.63 vs. M = 40.89, SD = 7.67, *t*(512) = 3.18, *p* = 0.002, Cohen’s *d* = 0.55).

Regarding the responses to the question, “What are your sources of information on sports nutrition?”, 478 participants reported obtaining nutritional information after their graduation. Frequency analysis revealed that 19.7% of participants obtained information from scientific papers, 41.2% from the internet, 4.8% from colleagues, 23.6% from dietitians/nutritionists, and 10.9% from seminars.

A one-way analysis of variance (ANOVA) was performed to examine differences in the total mean score across different sources of nutrition information. The analysis revealed that the differences between groups were not statistically significant (*F*(4, 473) = 2.301, *p* = 0.058), indicating that the source of nutrition information did not have a significant effect on the total mean score. The effect size, measured by partial eta squared, was small (η^2^_p_ *=* 0.019). Post hoc Bonferroni tests indicated no statistically significant pairwise differences between the groups (*p* > 0.050 for all comparisons). However, the highest mean scores were observed among participants who obtained information from nutrition seminars (M = 48.82, SD = 8.69), followed by those who consulted colleagues (M = 48.48, SD = 6.49) (Table 9).

## 4. Discussion

The present study aimed to translate, culturally adapt, and evaluate the psychometric properties of the Greek version of the Nutrition for Sport Knowledge Questionnaire (NSKQ-Gr) among Greek certified coaches. Overall, the findings indicate that the NSKQ-Gr demonstrated satisfactory psychometric properties, including good overall internal consistency and excellent temporal stability, supporting its use for assessing sports nutrition knowledge among Greek coaches. 

### 4.1. Psychometric Properties of the NSKQ-Gr

The present study substantially extends the psychometric evaluation of the NSKQ-Gr by combining complementary Classical Test Theory and Rasch approaches. Item difficulty analysis demonstrated that the questionnaire covers a broad spectrum of sports nutrition knowledge, including both very easy and very difficult items, supporting the instrument’s ability to differentiate respondents across varying levels of nutrition knowledge. Item discrimination, however, varied considerably across domains. Indeed, while the Alcohol domain demonstrated the strongest discrimination, Weight Management and Sports Nutrition contained a large number of items with low corrected item–total correlations, suggesting that the questionnaire might benefit from a refinement in future versions. 

The KR-20 and McDonald’s omega coefficients indicated acceptable internal consistency for most domains, with the exceptions of Weight Management and Sports Nutrition, a finding that is similar to those reported previously in Brazil [28], Jordan [44], and Germany [27], thus indicating that these domains consistently present greater psychometric challenges that are independent from the specific cultural settings. Importantly, lower internal consistency should not necessarily be interpreted as evidence of inadequate measurement. Knowledge questionnaires differ fundamentally from reflective psychological scales because their items are designed to assess complementary aspects of knowledge rather than interchangeable manifestations of a single latent construct. Consequently, lower internal consistency is expected for multidomain knowledge assessments in which content coverage is prioritized over item homogeneity.

The CFA findings provided mixed support for the proposed domain structure, with variability in both factor loadings and model fit across domains. These findings should be interpreted within the context of a multidomain knowledge assessment rather than a reflective latent variable model. Rather than indicating poor questionnaire quality, the results suggest that the NSKQ domains are better conceptualized as collections of related knowledge components than as strictly reflective one-factor constructs.

The Rasch analyses further complemented the Classical Test Theory findings. Even though Andersen likelihood-ratio tests were significant, most items demonstrated acceptable infit and outfit. The satisfactory item-level functioning suggests that the lack of complete Rasch model invariance is more likely attributable to domain heterogeneity than widespread item misfit. Interestingly, several of the items identified as problematic through corrected item–total correlations also demonstrated weaker Rasch fit, supporting the notion of further refinement of the instrument. 

Finally, the questionnaire demonstrated excellent temporal stability. Although this finding indicates very high test–retest reliability, values approaching unity should be interpreted cautiously because they may partly reflect methodological factors, including limited response variability and the relatively short retest interval (10 days). Nevertheless, the selected interval consists of methodological recommendations for questionnaire validation studies. Previous research suggests that a retest interval of approximately 1–2 weeks represents an appropriate balance between minimizing recall bias and avoiding genuine changes in the construct being measured. Moreover, Marx et al. [45] demonstrated that test–retest reliability did not differ significantly between assessments conducted after 2 days and after 2 weeks, supporting the use of intervals within this range. Similarly, the COSMIN guidelines [46,47] recommend that the retest interval should be sufficiently short to ensure construct stability while being long enough to reduce memory effects. Furthermore, the exceptionally high ICC observed in the present study may also reflect the expected stability of nutrition knowledge over a short period in the absence of any educational intervention. Nevertheless, future studies should evaluate the temporal stability of the NSKQ-Gr over longer retest intervals and in more heterogeneous samples to further confirm its long-term stability and reproducibility.

### 4.2. General Sports Nutrition Knowledge Levels

Using the classification described by Trakman et al. [26], the findings revealed a substantial gap in sports nutrition knowledge among Greek coaches, with the majority demonstrating only average or poor understanding and only a very limited proportion exhibiting advanced knowledge. These findings are consistent with previous studies reporting inadequate sports nutrition knowledge among coaches and athletes. Given that the participants in the present study were coaches of adolescent athletes (14–17 years), these findings are of particular importance. In youth sport, coaches play a primarily educational and supportive role and should not be regarded as substitutes for qualified sports dietitians or nutrition professionals. Rather, they should possess sufficient sports nutrition literacy to reinforce evidence-based practices, identify situations requiring specialist input, and refer to athletes when appropriate. This is especially relevant in the Greek youth sport setting, where access to sports dietitians may often be limited, particularly in community and semi-urban sports clubs. Therefore, improving the nutrition literacy of youth coaches through structured educational programs could complement the work of nutrition professionals and contribute to a more supportive environment for adolescent athletes, without replacing the role of specialized practitioners.

An interesting finding of the present study was that the reported sources of nutrition information were not significantly associated with sports nutrition knowledge. The prevalence of inadequate knowledge may reflect the absence of mandatory nutrition education within coach certification pathways, as well as reliance on informal or non-evidence-based information sources. As learning practices may play a significant role in shaping coaches’ nutrition knowledge, the absence of significant differences between sources of nutrition information suggests that access to information alone may be insufficient to enhance coaches’ overall sports nutrition knowledge. Even when coaches report consulting qualified professionals, attending nutrition seminars, or reading scientific articles, the lack of differentiation in knowledge scores may reflect differences in the depth of engagement with educational resources or difficulties in translating information into retained knowledge. Additionally, the quality, duration, and educational structure of seminars and professional consultations may vary considerably, potentially reducing their effectiveness. The slightly higher mean scores observed among coaches who attended nutrition seminars or relied on colleagues may indicate the value of experiential and socially mediated learning; however, without structured, evidence-based educational frameworks, such learning appears insufficient to produce substantial and consistent improvements in knowledge. 

While it is theoretically expected that higher levels of nutritional knowledge would be associated with increased provision of nutritional guidance to athletes, the results show that this relationship is not as strong as anticipated. Although a tendency toward greater involvement in providing nutritional advice is observed among coaches with higher knowledge scores, the associations are not strong, indicating that sports nutrition knowledge alone may not be sufficient to explain coaches’ provision of nutrition advice. Moreover, the provision of nutritional guidance may be influenced by external factors, such as perceived professional role boundaries, ethical considerations, and access to interdisciplinary support, which are not fully captured through the assessment of nutritional knowledge.

In investigating demographic and professional characteristics of coaches, the findings suggest that structural and educational factors may play a greater role than experiential factors in shaping coaches’ sports nutrition knowledge. Particularly, coaching experience was not significantly associated with NSKQ-Gr scores. Although coaches with more than 11 years of experience demonstrated slightly higher mean scores, the extremely small effect size indicates negligible practical significance. These findings indicate that coaching experience alone may be insufficient to ensure adequate nutrition competence without structured educational reinforcement. Conversely, the certification pathway was significantly associated with higher NSKQ-GR scores, with coaches certified by the Sport Sciences Departments achieving higher scores than those holding alternative certifications. Although this association was statistically significant, the observed effect size was small to moderate and should be interpreted with caution. Given the cross-sectional design of the study, these findings do not imply that certification itself led to higher sports nutrition knowledge. Instead, they suggest that the certification pathway may be associated with differences in nutrition knowledge or reflect differences in educational background, professional development opportunities, or individual motivation. Nevertheless, the findings support the potential value of incorporating evidence-based sports nutrition education into coaching certification programs and continuing professional development initiatives. Similarly, former athletic status was significantly associated with higher NSKQ-Gr scores, reflecting a moderate-to-large effect. This may indicate that experiential exposure within competitive sport environments facilitates deeper understanding [7,13,48]. In contrast, Miyazaki et al. [49] reported that previous participation in sport was not significantly associated with nutrition knowledge. However, relying on previous athletic experience as a pathway to nutrition competence may raise concerns regarding equitable access to nutrition knowledge. 

Finally, coaching as a primary livelihood was not associated with NSKQ-Gr scores. This suggests that professional engagement intensity does not inherently translate into improved nutrition competence. Working full-time as a coach does not necessarily ensure greater exposure to evidence-based nutrition knowledge.

Coaches’ age was also examined as a potential factor influencing sports nutrition knowledge. The results revealed a significant difference in NSKQ-Gr scores across age groups, with coaches aged 53–62 years exhibiting the highest mean scores compared with younger age groups. This finding is consistent with previous research suggesting that experience and prolonged exposure to sports nutrition education positively influence knowledge levels [5,14,19,44]. Although younger coaches were expected to demonstrate higher levels of nutrition knowledge due to their more recent university education [16,23], this assumption was not supported by the present findings. Older coaches may have had more opportunities for professional development and practical experience, leading to a better understanding and application of nutrition principles. 

Our findings revealed no significant differences in sports nutrition knowledge between male and female coaches. Similar findings have been reported by Couture et al. [19] among high school coaches, Spendlove et al. [50] among athletes, and Miyazaki et al. [49] among Japanese coaches. However, the categorical analysis identified differences in the distribution of participants across the predefined knowledge categories. This apparent discrepancy likely reflects the use of predefined classification thresholds rather than meaningful differences in overall sports nutrition knowledge and should therefore be interpreted with caution. It may also reflect differences in the distribution of scores around the predefined category cut-off points. Further research is warranted to explore potential factors that may influence sports nutrition knowledge, including educational opportunities, access to nutrition information, engagement in continuing professional development, and individual interest in sports nutrition.

Coaches’ BMI classifications did not significantly influence NSKQ scores. Existing studies that explore the relation between nutritional knowledge and body weight (or obesity) in different populations did not reveal a significant connection. Most of them assert that social, economic, and other factors play a role in shaping the outcomes [51,52,53,54]. These findings are consistent with previous studies reporting no significant association between BMI and nutrition knowledge across different populations, suggesting that body weight is influenced by multiple behavioral, environmental, and socioeconomic factors beyond nutrition knowledge alone [48,55,56,57]. 

### 4.3. Study Strengths and Limitations

This study has several important strengths. It used an internationally validated instrument, facilitating cross-cultural comparisons, and included a large and diverse sample of 514 certified coaches from multiple sports disciplines, enhancing the representativeness of the findings within the Greek coaching population, while the translation and cultural adaptation of the NSKQ, which followed internationally recognized forward–backward procedures and involved expert evaluation, strengthened content validity. This study, based on the complementary implementation of Classical Test Theory and Rasch approaches, provides a more comprehensive psychometric evaluation than previously available for the Greek version of the NSKQ, while the excellent test–retest reliability and temporal stability suggest that the NSKQ-Gr is a suitable instrument for assessing nutrition-related knowledge among Greek coaches. Finally, the large number of demographic, professional, and nutrition-related practice variables that were included provided a broader understanding of factors associated with sports nutrition knowledge among coaches.

However, certain limitations should be acknowledged. The cross-sectional design of this study precludes causal inferences; therefore, the observed associations should not be interpreted as evidence of cause-and-effect relationships. The voluntary online recruitment and convenience sampling approach may also have introduced selection bias, as coaches with a greater interest in sports nutrition or continuing professional education may have been more likely to participate, thus potentially limiting generalizability. 

From a psychometric perspective, although the overall findings support the adequacy of the NSKQ-Gr, some domains demonstrated lower internal consistency and weaker item discrimination. These findings are indicative of the need to further refine the instrument. Measurement invariance and differential item functioning analyses can be of great assistance in future endeavors. 

Additionally, this study assessed whether coaches provided nutritional advice but did not evaluate the quality or accuracy of that advice. In addition, although the 10-day interval used to assess test–retest reliability and categorical agreement is consistent with previous reliability studies, it may have increased the possibility of recall effects, potentially contributing to the exceptionally high reliability estimates observed. 

Finally, a limitation of the present study is that the questionnaire did not specifically distinguish AI-based tools (e.g., AI chatbots or AI-assisted search tools) from other internet-based sources of nutrition information. This approach was adopted to maintain fidelity to the original NSKQ and preserve its conceptual and structural equivalence throughout the cross-cultural adaptation process. Furthermore, at the time of data collection, AI-based tools had not yet become as widely integrated into educational and professional practice as they are today. 

## 5. Conclusions

In conclusion, the present study provides the first comprehensive cross-cultural adaptation and psychometric evaluation of the Greek version of the Nutrition for Sport Knowledge Questionnaire (NSKQ-Gr) in certified Greek coaches. 

The findings also indicate that most Greek coaches demonstrated only average or poor sports nutrition knowledge, highlighting the need for structured educational programs within coach education and continuing professional development, particularly for coaches working with children and adolescent athletes, whose influence on the development of dietary habits and health-related behaviors is especially important. Such initiatives should aim to enhance coaches’ nutrition literacy and facilitate effective collaboration with qualified sports dietitians rather than replace their professional role. 

## Figures and Tables

**Table 1:** Descriptive statistics for anthropometric data of the coaches.

	Age (Years)	Weight (kg)	Height (m)	BMI (kg/m^2^)
Male ^1^	34.89 (±9.09)	84.11(±11.31)	1.80 (±0.07)	25.73 (±2.98)
Female ^1^	34.41 (±9.23)	67.46(±12.61)	1.70 (±0.06)	23.33 (±3.59)
Total ^1^	35.10 (±9.15)	77.43(±14.38)	1.76 (±0.09)	24.77 (±3.44)

^1^ Data are presented as mean ± standard deviation (M ± SD).

**Table 2:** Mean percentage (%) among knowledge categories by sex and sport type.

Knowledge Category	All Coaches (*n* = 514)	Male (*n* = 308)	Female (*n* = 206)	Team Sports (*n* = 236)	Individual Sports (*n* = 278)
Poor	32.3	28.6	37.9	33.9	30.9
Average	56.8	60.4	51.5	54.2	59.0
Good	8.6	11.0	4.9	10.2	7.2
Excellent	2.3	0.0	5.8	1.7	2.9

**Table 3:** Descriptive statistics and Pearson correlations among the NSKQ domains.

NSKQ-Gr Domains	Mean	SD	2	3	4	5	6
1. Weight Management	6.68	1.86	0.536 **	0.366 **	0.362 **	0.346 **	0.081
2. Macronutrients	15.80	4.37	—	0.485 **	0.427 **	0.480 **	0.153 **
3. Micronutrients	6.57	2.21	—	—	0.349 **	0.380 **	0.041
4. Sports Nutrition	5.10	1.71	—	—	—	0.338 **	0.051
5. Supplementation	4.72	2.99	—	—	—	—	0.075
6. Alcohol	6.78	1.31	—	—	—	—	—

Values are Pearson correlation coefficients (*r*). ** *p* < 0.01 level (2-tailed). SD = standard deviation. *N* = 514.

**Table 4:** Most difficult and easiest NSKQ-Gr items according to the proportion of correct responses.

Most Difficult Items	% Correct	Easiest Items	% Correct
NSKQ58. Athletes should drink water to:	4.3	NSKQ80. How much ethanol (pure alcohol) is there in a standard drink?	100.0 *
NSKQ56. How much sodium (salt) should fluid consumed for hydration purposes (during exercise) contain?	5.1	NSKQ85. Drinking lots of alcohol can make it harder to recover from injury	100.0 *
NSKQ67. How much protein do you think experts say athletes should eat after resistance exercise?	10.1	NSKQ10. To ensure they meet their energy (kilojoule/calorie) requirements, all athletes should	96.1
NSKQ53. Athletes aged 15 to 24 years need 500 mg of calcium each day.	11.7	NSKQ82. Do you think alcohol can make you put on weight?	94.9
NSKQ63. Some athletes get a sore stomach if they eat during exercise. What might make stomach pain worse?	12.5	NSKQ86. Do you agree or disagree with the statement: Alcohol makes you urinate more.	94.9
		NSKQ81. Which is an example of a “Standard Drink”?	93.8
		NSKQ7. Choosing lower glycemic index (GI) carbohydrates to help regulate appetite	89.9
		NSKQ23. Which of these foods do you think have enough protein to promote muscle growth after a bout of resistance exercise? 300 gr yellow cheese	89.9

* Items NSKQ80 and NSKQ85 exhibited zero response variability (100% correct responses).

**Table 5:** Summary of item difficulty and discrimination indices across the six NSKQ-Gr domains.

Domain	*n*	Mean Difficulty	Difficulty Range	Mean Corrected Item–Total	Items < 0.20
Weight Management	12	0.557	0.218–0.961	0.109	10
Macronutrients	30	0.530	0.156–0.899	0.208	13
Micronutrients	13	0.506	0.117–0.875	0.236	7
Sports Nutrition	12	0.425	0.043–0.739	0.094	10
Nutritional Supplements	12	0.394	0.179–0.794	0.236	4
Alcohol *	6	0.797	0.607–0.949	0.357	1

* Items NSKQ80 and NSKQ85 were not included because they showed zero response variability (100% correct answers).

**Table 6:** Chi-square test results for group differences.

Knowledge Level	Male (%)	Female (%)	*χ*^2^, *p*, *V*	Team Sports (%)	Individual Sports (%)	*χ*^2^, *p*, *V*
Poor	28.6	37.9	*χ*^2^ = 28.49(3), *p* < 0.001, *V* = 0.235	33.9	30.9	*χ*^2^ = 2.94(3), *p* = 0.401, *V* = 0.076
Average	60.4	51.5		54.2	59	
Good	11	4.9		10.2	7.2	
Excellent	0	5.8		1.7	2.9	

**Table 7:** Comparison of total NSKQ-Gr scores according to demographic and professional characteristics.

Characteristic	Category	Total NSKQ-Gr Score (M ± SD)	Test Statistic	*p*	Effect Size
Age	22–32 years	44.67 ± 9.37	*F*(3, 510) = 3.839	0.010	η^2^_p_ *=* 0.022
	33–42 years	46.89 ± 9.98			
	43–52 years	45.30 ± 10.44			
	53–62 years	49.87 ± 4.17			
Sex	Male	46.13 ± 9.87	*t*(512) = 1.095	0.274	*d* = 0.10
	Female	45.18 ± 9.14			
BMI (kg/m^2^)	Underweight	45.50 ± 13.27	*F*(3, 510) = 0.192	0.902	η^2^_p_ = 0.001
	Normal weight	45.76 ± 9.33			
	Overweight	45.52 ± 9.73			
	Obese	46.76 ± 10.56			
Coaching experience	2–5 years	44.84 ± 9.62	*F*(2, 511) = 0.911	0.149	η^2^_p_ = 0.007
	6–10 years	45.12 ± 9.96			
	>11 years	46.66 ± 9.25			
Certification pathway	DPESS	47.08 ± 8.37	*U =* 25,106	<0.001	*r =* 0.15
	Other	43.44 ± 11.05			
Former athlete	Yes	46.18 ± 9.83	*U =* 5586	<0.001	*r =* 0.20
	No	40.95 ± 3.98			
Level of competition	National	45.67 ± 9.87	*F*(2, 511) = 1.815	0.164	η^2^_p_ = 0.008
	European/International	47.74 ± 9.51			
	Local	47.03 ± 10.03			
Coaching as primary occupation	Yes	46.41 ± 8.48	*U* = 32,022	0.596	*r* = 0.02
	No	45.00 ± 10.70			

Values are presented as mean ± standard deviation (SD). One-way ANOVA results are reported as *F* statistics with partial eta squared (η^2^_p_ ) as the corresponding effect size. Independent-samples *t*-tests are reported with Cohen’s *d*, whereas Mann–Whitney *U* tests are reported with effect size *r*. *p* < 0.001 is reported for very small *p*-values. DPHSS: Department of Physical Education and Sports Science.

**Table 8:** Comparison of total ΝSKQ-Gr scores according to coaches’ nutrition-related practices.

Nutrition-Related Practice	Category	Mean ± SD	Test Statistic	*p*-Value	Effect Size
Have you provided nutritional information to your athletes?	Yes	46.75 ± 8.60	*U* = 6834	<0.001	*r* = 0.24
	No	37.26 ± 12.93			
How often do you provide nutritional advice?	1–2/week	50.84 ± 9.80	*F*(2, 511) = 15.63	<0.001	η^2^_p_ = 0.065
	1–2/month	45.82 ± 7.70			
	Only if asked	46.57 ± 8.50			
Do you weigh your athletes?	Yes	47.86 ± 9.14	*U =* 24,030	<0.001	*r* = 0.23
	No	43.41 ± 9.54			
Are you informed about nutritional issues?	Yes	46.12 ± 9.63	*t*(512) = 3.18	0.002	*d* = 0.55
	No	40.89 ± 7.67			

Note. Test statistic is reported as *F* for one-way ANOVA, *t* for independent-samples *t*-tests, and *U* for Mann–Whitney U tests. Effect sizes are reported as partial η^2^, Cohen’s *d,* or r, respectively.

**Table 9:** Total NSKQ-Gr scores according to sources of nutrition information.

			95% Confidence Interval	
Source of Information	Mean	SD	Lower for Mean	Upper for Mean
Scientific Papers	45.83	9.26	43.93	47.73
Internet	44.91	9.97	43.51	46.31
Colleagues	48.48	6.49	45.67	51.29
Dietitians	46.76	10.01	44.90	48.63
Nutrition Seminar	48.82	8.69	46.38	51.27
Total	46.12	9.63	45.25	46.98

## Data Availability

Data are available upon request to the corresponding authors.

## Appendix A

**Table A1:** Item-level psychometric characteristics of the NSKQ-Gr.

Domain	Item	Difficulty (% Correct)	Corrected Item–Total	Infit	Outfit
Weight Management	NSKQ1	29.6	0.186	0.900	0.850
	NSKQ2	44.7	0.052	1.037	1.088
	NSKQ3	21.8	−0.011	1.041	0.995
	NSKQ4	54.9	0.178	0.942	0.945
	NSKQ5	66.9	−0.102	1.161	1.205
	NSKQ6	39.7	0.075	1.011	0.981
	NSKQ7	89.9	0.140	0.845	0.835
	NSKQ8	68.5	0.145	0.970	0.913
	NSKQ9	47.5	0.180	0.944	0.889
	NSKQ10	96.1	−0.052	0.964	1.397
	NSKQ11	42	0.207	0.916	0.878
	NSKQ12	66.9	0.311	0.848	0.755
Macronutrients	NSKQ13	45.5	0.228	0.985	0.996
	NSKQ14	56	0.082	1.086	1.169
	NSKQ15	15.6	−0.149	1.172	1.339
	NSKQ16	85.2	0.305	0.901	0.803
	NSKQ17	64.2	0.306	0.936	0.898
	NSKQ18	31.1	0.098	1.042	1.046
	NSKQ19	4.5	0.034	1.123	1.144
	NSKQ20	31.9	0.342	0.882	0.849
	NSKQ21	42.8	0.346	0.901	0.872
	NSKQ22	52.5	0.263	0.966	0.929
	NSKQ23	89.9	0.179	0.950	1.039
	NSKQ24	51	0.024	1.132	1.116
	NSKQ25	51.8	0.177	1.025	0.992
	NSKQ26	75.5	0.059	1.103	1.083
	NSKQ27	54.1	0.375	0.890	0.862
	NSKQ28	56	0.023	1.132	1.502
	NSKQ29	44.4	0.194	1.008	0.981
	NSKQ30	39.3	0.282	0.938	0.916
	NSKQ31	73.9	0.264	0.944	0.939
	NSKQ32	54.5	0.370	0.893	0.867
	NSKQ33	47.5	0.308	0.934	0.940
	NSKQ34	76.7	0.181	0.995	0.998
	NSKQ35	49	0.061	1.105	1.083
	NSKQ36	46.3	0.234	0.985	0.947
	NSKQ37	52.1	0.320	0.927	0.891
	NSKQ38	59.1	0.312	0.933	0.898
	NSKQ39	70	0.200	0.995	0.991
	NSKQ40	28.8	0.278	0.942	0.903
	NSKQ41	39.7	0.176	1.017	0.985
	NSKQ42	63.8	0.353	0.912	0.854
	NSKQ43	87.5	0.186	0.920	1.104
	NSKQ44	8.9	0.148	1.054	0.962
	NSKQ45	13.6	0.207	0.891	0.726
	NSKQ46	16.3	0.256	0.848	0.811
	NSKQ47	75.1	0.390	0.800	0.741
	NSKQ48	67.3	0.138	1.044	1.284
	NSKQ49	44	0.424	0.800	0.740
	NSKQ50	51.8	0.170	1.061	1.067
	NSKQ51	53.7	0.164	1.071	1.080
	NSKQ52	85.6	0.353	0.772	0.705
	NSKQ53	11.7	0.303	0.728	0.641
	NSKQ54	17.1	0.167	0.922	1.043
	NSKQ55	52.9	0.160	1.059	1.159
Sports Nutrition	NSKQ56	5.1	−0.130	0.901	1.451
	NSKQ57	73.9	0.093	0.989	0.984
	NSKQ58	4.3	0.050	0.814	0.643
	NSKQ59	61.1	0.013	1.069	1.078
	NSKQ60	52.1	0.218	0.904	0.865
	NSKQ61	34.2	0.069	0.989	0.994
	NSKQ62	73.2	0.151	0.937	0.915
	NSKQ63	12.5	0.099	0.859	0.796
	NSKQ64	64.6	0.304	0.834	0.787
	NSKQ65	61.5	0.131	0.974	0.961
	NSKQ66	57.2	0.048	1.041	1.058
	NSKQ67	1.1	0.079	0.835	0.775
Nutritional Supplements	NSKQ68	52.9	0.225	1.008	0.970
	NSKQ69	23.7	0.357	0.844	0.713
	NSKQ70	31.1	0.190	1.019	1.144
	NSKQ71	36.2	0.351	0.886	0.829
	NSKQ72	27.2	0.216	0.974	0.967
	NSKQ73	58.8	0.016	1.247	1.259
	NSKQ74	42.8	0.354	0.892	0.829
	NSKQ75	47.5	0.385	0.867	0.807
	NSKQ76	25.3	0.226	0.962	0.924
	NSKQ77	29.6	0.094	1.113	1.223
	NSKQ78	17.9	0.199	0.997	0.755
	NSKQ79	79.4	0.223	0.920	0.880
Alcohol	NSKQ80 *	10.0	NA	-	-
	NSKQ81	93.8	0.096	0.897	1.750
	NSKQ82	94.9	0.256	0.684	0.408
	NSKQ83	65.4	0.640	0.670	0.604
	NSKQ84	6.7	0.228	1.408	1.894
	NSKQ85 *	10.0	NA	-	-
	NSKQ86 **	94.9	0.256	-	-
	NSKQ87	68.5	0.668	0.608	0.554

Note. Difficulty is expressed as the percentage of participants answering each item correctly. Corrected item–total correlations were calculated within each theoretical domain. Standardized CFA loadings were obtained from the domain-specific confirmatory factor analyses. Rasch item fit is presented as infit and outfit mean-square statistics (MNSQ). Values between 0.70 and 1.30 indicate acceptable item fit. * NSKQ80 and NSKQ85 showed zero response variability, and, therefore, corrected item–total correlations and Rasch statistics were not interpretable. ** NSKQ86 showed an identical response pattern to NSKQ82 and was therefore not estimated separately in the Rasch analysis.
